# Early‐Life Ceftriaxone‐Induced Gut Microbiota Perturbation Persistently Exacerbates Juvenile ADHD‐Like Behaviours via Immune Dysfunction in SHR/WKY Rats

**DOI:** 10.1111/1751-7915.70255

**Published:** 2025-10-23

**Authors:** Yang Yang, Simou Wu, Jianxiu Liu, Kai Wang, Yating Luo, Jinxing Li, Zhimo Zhou, Fang He, Ruyue Cheng

**Affiliations:** ^1^ Department of Nutrition and Food Hygiene West China School of Public Health and West China Fourth Hospital, Sichuan University Chengdu P.R. China

**Keywords:** attention deficit hyperactivity disorder (ADHD), ceftriaxone sodium, early life, gut microbiota, inflammation

## Abstract

This study investigated the impact of ceftriaxone‐induced gut microbiota perturbation in neonatal male spontaneous hypertensive rats (SHR) and Wistar‐Kyoto (WKY) rats during lactation on the development of juvenile ADHD symptoms. The 5‐choice serial reaction time task (5‐CSRTT) and open‐field test (OFT) were used to evaluate ADHD‐related behaviours, and alterations in immune pathways within the microbiota‐gut‐brain axis were examined. At 3 weeks old, the gut microbiota in both WKY and SHR was significantly disrupted following antibiotic intervention, with these changes persisting 4 weeks after ceftriaxone withdrawal. At the juvenile stage, WKY exhibited inattention, impulsivity, and hyperactivity, while SHR had severe hyperactivity and neuroinflammation. Decreased Chao1 and Shannon indices were positively associated with Treg cells in the spleen, mesenteric lymph nodes (MLN), and IL‐10 mRNA expression in the striatum; further, the latter biochemical indices were negatively associated with ADHD symptoms. *Lactobacillus* and *Clostridia_UCG‐014* negatively correlated with Treg cells in the spleen, MLN, IL‐6, and IL‐10 mRNA expression in the striatum; further, these biomarkers were negatively associated with ADHD, which suggested they may contribute to the development of ADHD. In contrast, *Muribaculaceae* positively correlated with Treg cells in the spleen and MLN, IL‐10 mRNA expression, and negatively correlated with ADHD symptoms. These results suggest that early life gut microbiota perturbation persistently contributes to the onset and aggravation of juvenile ADHD through the exacerbation of neuroinflammation and peripheral immune dysfunction.

## Graphical abstract

After early life gut dysbiosis by antibiotic, SHR had a sever hyperactivity and neuroinflammation, and WKY became inattentive, impulsive and more hyperactive in juvenile.

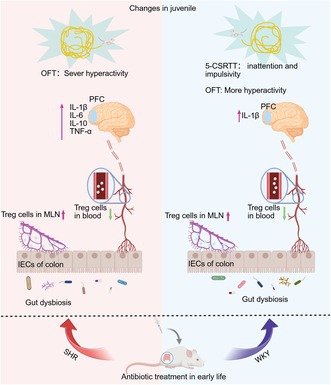

## 1 Introduction

Early life is widely recognised as a critical period for the establishment of the gut microbiota, which significantly influences host health in later stages of life, consistent with the Developmental Origins of Health and Disease (DOHaD) theory (Barker 2004). Disruptions to gut microbiota colonisation during this window may increase susceptibility to a range of diseases in later life. Ceftriaxone, a third‐generation cephalosporin antibiotic, is commonly used to induce early life gut dysbiosis due to its poor intestinal absorption (Cho et al. 2004) and widespread use in paediatric care (Wang et al. 2020). Previous studies have shown that ceftriaxone‐induced gut dysbiosis during early life can elevate the risks of colitis, allergic diseases, and obesity (Peng et al. 2022; Cheng et al. 2019; Miao et al. 2021). Recent evidence also highlights the associations between early life gut microbial composition and nervous system development, primarily based on studies employing germ‐free animal models or antibiotics‐induced microbiota depletion in mice (Clarke et al. 2013; Lynch et al. 2023).

Attention‐deficit/hyperactivity disorder (ADHD) is a common neurodevelopmental disorder that primarily affects children and adolescents, characterised by inattention, impulsivity, and hyperactivity, with a higher prevalence in males, according to the *Diagnostic and Statistical Manual of Mental Disorders*, Fifth Edition (DSM‐5). The microbiota‐gut‐brain (MGB) axis, a bidirectional communication system linking the gut and the brain through neuronal, endocrine, and immune pathways (Agirman and Hsiao 2021), has been proposed to play a potential role in the aetiology of ADHD. Recent research suggests that early life gut microbiota dysbiosis, arising from factors such as infections, antibiotic exposure, stress, and prenatal conditions, may increase the risk of developing ADHD and other neurodevelopmental disorders (Ahrens et al. 2024). However, the specific mechanisms through which the MGB axis mediates the association between early life gut microbiota perturbations and ADHD remain unclear, highlighting the need for mechanistic investigations employing experimental approaches.

Spontaneous hypertensive rats (SHR) are commonly employed as an animal model of ADHD, typically using Wistar‐Kyoto (WKY) rats as controls. SHR display core ADHD‐like behaviors, including hyperactivity, inattention, and impulsivity, that closely parallel the clinical manifestation of the disorder (Sagvolden et al. 1992; Jentsch 2005). Emerging evidence indicates that SHR exhibit heightened neuroinflammatory responses, as demonstrated by higher levels of activated microglia and TNF‐α in the brain (Fang et al. 2023). Neuroinflammation has been increasingly recognized as a potential risk factor and contributing mechanism in ADHD pathophysiology (Dunn et al. 2019). However, the effects of antibiotic‐induced early life gut microbiota perturbations on neuroinflammation and ADHD‐like behaviors in juvenile SHR remain largely unexplored.

Therefore, this study utilises ceftriaxone sodium to induce gut microbiota perturbations in postnatal male SHR and WKY, aiming to assess the subsequent effects on ADHD‐like behaviours and exploring potential underlying mechanisms, thereby leading to a better understanding of the causal relationship between gut microbiota and ADHD.

## 2 Materials and Methods

### 2.1 Animal Breeding

The animal experimental protocols were approved by the Ethics Committee of West China School of Public Health and West China Fourth Hospital, Sichuan University (ethical approval code: Gwll2022057). 10‐week‐old male and female SHR/Ncrl and WKY/Ncrl rats were purchased from Beijing Vital River Laboratory Animal Technology Co. Ltd. (Beijing, China), and subjected to a 1‐week acclimatisation period.

After mating and pregnancy, the newborn male pups were selected for this experiment. During the first 3 weeks postnatally, pups received daily oral gavage of either saline solution (Control group) or ceftriaxone sodium (Abx group, 50 mg/kg body weight [BW]). After weaning at 3 weeks of age, the gavage was discontinued. Afterwards, the 5‐choice serial reaction time task (5‐CSRTT) and open field test (OFT) were conducted successively. At 7 weeks, the rats were sacrificed after being euthanized under anaesthesia with Zoletil 50 (10 mg/kg BW).

### 2.2 5‐CSRTT

The traditional 5‐CSRTT usually spans about 1 month or longer. However, the adolescent period in rodents is too short to accommodate the standard protocol. Hence, modified versions of the 5‐CSRTT, lasting one or two weeks, have been developed for adolescent mice (Remmelink et al. 2017; Ciampoli et al. 2017). In this study, we implemented a modified 5‐CSRTT protocol lasting about 3 weeks, adapted for juvenile SHR and WKY, based on the methods described by Bari et al. (2008) and Ciampoli et al. (2017).

The operating chamber is manufactured by Shanghai Xinruan Information Technology Co. Ltd. (Shanghai, China). The modified 5‐CSRTT consisted of 3 adaptation days, 16 training days, and 1 test day. Each day, rats were randomly placed in the chamber. Animals were food‐restricted and allowed ad libitum access to food for 3 h after each session, while water was available ad libitum throughout the experiment.

During the adaptation days, rats were placed in the chamber for 15–20 min every day, and body weight was maintained at 80% to 90% of baseline to enhance motivation.

During training, each session began with a 5 s intertrial interval (ITI), and a nose‐poke during this period was recorded as a premature response, leading to a 1 s loudspeaker stimulus on and a 5 s timeout period during which the house light was turned off. After ITI, one of 5 holes was illuminated for a specific duration (stimulus duration, SD), followed by a limited hold (LH) period. A nose‐poke into the right, illuminated hole during SD or LH was recorded as a correct response with a food pellet reward delivered to the food magazine. Otherwise, nose‐pokes into the wrong hole in the SD and LH period were recorded as incorrect responses, resulting in a 1 s loudspeaker stimulus on and a 5 s timeout period with no food reward. A failure to respond during the SD and LH period was recorded as an omission and followed by punishment. In addition, nose‐pokes made after a correct response, unless directed toward the food magazine, were recorded as perseverative responses. Nose‐pokes occurring during the timeout period were recorded as a timeout response. Each daily training session concluded when a rat completed 100 trials or reached a duration of 20 min. The durations of SD, LH, ITI, and timeout were listed in Table S1.

After the test day, data from the rats with omission (%) that were no more than 60% were included in the analysis. Behavioural measures were recorded and calculated as follows.

Omission (%): the number of omissions divided by the total number of trials, multiplied by 100.

Accuracy (%): the number of correct responses divided by the sum of correct and incorrect responses, multiplied by 100.

Correct response (%): the number of correct responses divided by the total number of trials, multiplied by 100.

Incorrect response (%): the number of incorrect responses divided by the total number of trials, multiplied by 100.

Premature responses: number of premature responses.

### 2.3 OFT

The OFT was used to evaluate hyperactivity. The OFT equipment used for the test was manufactured by Chengdu Techman Software Co. Ltd. (Chengdu, China). The open field measured 50 × 50 cm, and was divided into 9 zones, with 1 central zone, 4 corner zones and 4 side zones. Each rat was put into the central zone, and the test lasted 5 min. The time moving and distance moved were recorded and analysed for each rat. After each session, 75% (v/v) alcohol was used to eliminate odour and residual traces.

### 2.4 Detection of the Gut Microbiota Composition by 16S rRNA Sequencing

Faecal samples were collected at weaning (3 weeks of age) and before euthanization (7 weeks of age) and then frozen at −80°C immediately. For the 3‐week timepoint, 5 mixed faecal samples of 100 mg in each group were prepared, while at 7 weeks, individual faecal samples of 100 mg were prepared. After DNA extraction, amplification of the V3–V4 region by polymerase chain reaction (PCR), quantitation of PCR products, establishment of an Illumina library, and Illumina sequencing, the original sequences were obtained and denoised with DADA2 or Deblur in QIIME2 to generate amplicon sequence variant (ASV). Microbial composition, diversity, and variation were analysed based on the resulting ASV table. α diversity and principal coordinate analysis (PCoA) of β diversity were calculated on the Meiji online platform (https://cloud.majorbio.com). Relative abundance of microbial taxa was calculated using R 4.2.2.

### 2.5 Reverse Transcription Real‐Time Quantitative PCR

Total RNA was extracted from the colon, prefrontal cortex, striatum, hippocampus, and midbrain using the Animal Total RNA Isolation Kit (Chengdu Foregene Biotech Co. Ltd., Chengdu, China), following the manufacturer's instructions. Complementary DNA (cDNA) was then synthesized using the iScript cDNA Synthesis Kit (Bio‐Rad Laboratories Inc., Hercules, CA, USA). qPCR was performed using SsoAdvanced Universal SYBR Green Supermix (Bio‐Rad Laboratories Inc.) and the ABI QuantStudio 3 system (Thermo Fisher Scientific, Massachusetts, USA).

The relative mRNA expression levels of the targeted genes were subsequently analysed, including Interleukin 1 beta (*Il1β*), Interleukin 6 (*Il6*), Interleukin 10 (*Il10*), Tumour necrosis factor (*Tnfα*). The geometric means of the cycle thresholds (CT) of Glyceraldehyde‐3‐phosphate dehydrogenase (*Gapdh*), Actin, beta (*Actb*), and Ribosomal protein lateral stalk subunit P0 (*Rplp0*) were calculated and used as internal reference values. Relative fold changes in targeted mRNA expression were calculated using the $2^{-\Delta \Delta \mathrm{CT}}$ method. Table S2 shows the reverse‐transcription PCR protocol. Table S3 shows the qPCR protocol. Table S4 shows the primer sequences used for qPCR.

### 2.6 Immunofluorescence (IF) Staining for Cytokines in the Prefrontal Cortex

The entire brains of the rats were fixed in 10% paraformaldehyde for 24–72 h, and embedded with paraffin. Prefrontal cortex sections underwent dewaxing, antigen retrieval, and sealing before incubation with anti‐IL‐1β antibody (1:100), anti‐IL‐6 antibody (1:100), anti‐IL‐10 antibody (1:100), or anti‐TNF‐α antibody (1:100) at 4°C overnight. The secondary antibody Alexa Fluor594 donkey anti‐rabbit lgG (H + L) (1:400) was then applied at 37°C for 45 min. After DAPI staining, the sections were examined under a microscope, and the ratios of IL‐1β, IL‐6, IL‐10, and TNF‐α positive cells to DAPI in 8 fields of 40‐fold magnification were calculated, respectively.

### 2.7 Flow Cytometry for Treg Cells Detection

Blood from the abdominal aorta was collected in a blood collection tube with EDTA‐2Na, and then mesenteric lymph nodes (MLN) and spleens were removed and dipped into phosphate‐buffered saline (PBS).

The samples were preprocessed first. After the addition of red blood cell lysate at room temperature for 10 min, blood samples were lysed and washed twice with PBS, after which the precipitates were collected after centrifugation. MLN and spleen tissues were cut into small pieces and filtered with a 200‐mesh cell sieve, followed by centrifugation at 300 × *g* for 5 min. Hence, the precipitates were retained and washed twice with PBS, followed by centrifugation at 300 × *g* for 5 min to obtain the precipitates. Afterwards, the precipitates were lysed in red blood cell lysate at room temperature for 10 min. Finally, the precipitates were washed twice with PBS and collected after centrifugation.

The precipitates were then used for staining and Treg detection. All the antibodies used were from Ebioscience. The precipitates of the blood, MLN, and spleen samples were resuspended in 100 μL of PBS, and CD4 (0.5 μL/case) and CD25 (0.625 μL/case) were added. The samples were incubated at 4°C for 30 min in the dark and centrifuged at 300 × *g* for 5 min, after which the supernatant was discarded. After washing with PBS and centrifuging to maintain precipitate formation, each sample was added with 500 μL of 1 Fixation/Permeabilization Concentrate, followed by incubation at room temperature for 50 min and centrifugation at 350 × *g* for 5 min; then the supernatant was discarded. Afterwards, each sample was washed twice with 250 μL of 1× permeabilization buffer, centrifuged at 350 × *g* for 5 min, and 100 μL of 1× permeabilization buffer and Foxp3 (5 μL/case) were added, and then the mixture was incubated at 4°C overnight, followed by centrifugation at 350 × *g* for 5 min to obtain the precipitates. Finally, the precipitates were washed with 250 μL of permeabilization buffer and centrifuged at 350 × *g* for 5 min, and the precipitates were resuspended in 300 μL of 1× true nuclear perm. The samples were then used to detect the number of CD4^+^CD25^+^Foxp^+^ cells. CytExpert was used to analyze the proportion of CD4^+^CD25^+^Foxp^+^ cells in CD4^+^ cells.

### 2.8 Statistical Analysis

The data are expressed as $\bar{x}\pm \mathrm{SEM}$ in GraphPad Prism 9. Two‐way ANOVA (species × intervention) or three‐way ANOVA (species × intervention × age) was conducted. For Pearson correlation analysis, the *r* and *p* values were calculated with the corr.test function in the psych package of R 4.2.2 and were visualised with Originlab. *p* < 0.05 was considered statistical significance.

## 3 Results

### 3.1 Ceftriaxone‐Induced Gut Microbiota Perturbation in Early Life Lasted to Juvenile

**FIGURE 1:**
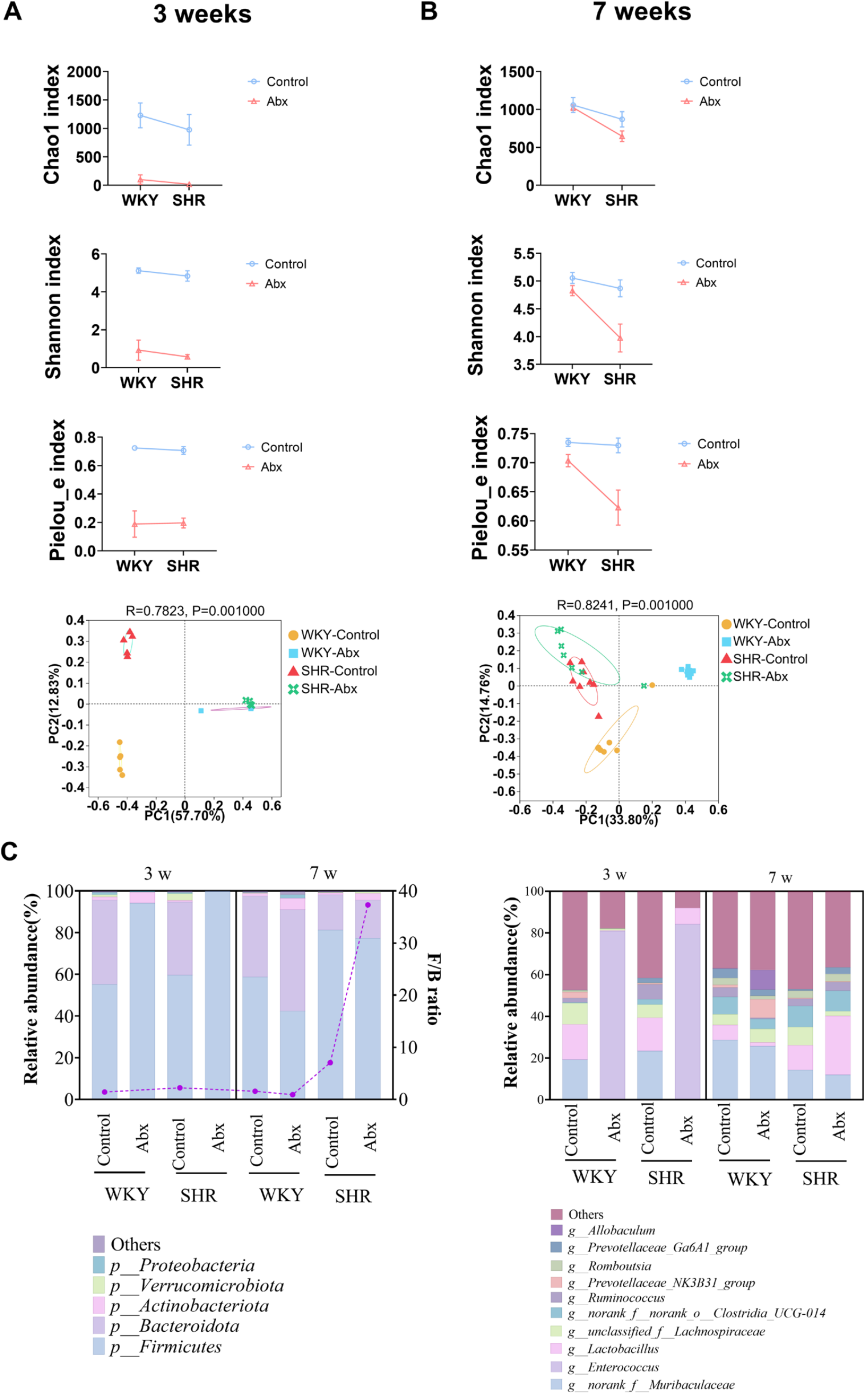
Effects of early life ceftriaxone intervention on gut microbiota. (A) α diversity and PCoA map of β diversity at 3 weeks. (B) α diversity and PCoA map of β diversity at 7 weeks. (C) Relative abundance of gut microbiota at phylum (left) and genus (right) levels. Abx, ceftriaxone group. The dashed line in the relative abundance at phylum level represents the F/B ratio. The F/B ratio was not marked in antibiotic groups at 3 weeks because the relative abundance of *Bacteroidota* was close to 0. *n* = 5 in each group at 3 weeks, *n* = 7–9 in each group at 7 weeks.

In α diversity indices (Chao1, Shannon, and Pielou_e indices), the effect of our intervention had interactions with age (Table 1). At 3 weeks, ceftriaxone administration significantly altered both α and β diversity of the gut microbiota in both WKY and SHR (Figure 1A). At 7 weeks, the Chao1 Index was lower in SHR compared to WKY. Furthermore, the decreased Shannon and Pielou_e indices in SHR, induced by ceftriaxone during weaning, persisted through 7 weeks of age, an effect not observed in WKY (Figure 1B). However, significant separation in β diversity was observed between WKY‐control and WKY‐Abx groups at 7 weeks, whereas this separation was not significant in SHR (Figure 1B).

**TABLE 1:** ANOVA table of α diversity indices.

Index	Source of variation	Df	*F*	*p*
Chao1 Index	Age	1	29.63	< 0.001
	Species	1	14.74	< 0.001
	Intervention	1	99.76	< 0.001
	Age × species	1	0.96	0.332
	Age × intervention	1	60.66	< 0.001
	Species × intervention	1	0.01	0.937
	Age × species × intervention	1	2.38	0.131
	Residual	43		
Shannon Index	Age	1	134.30	< 0.001
	Species	1	7.09	0.011
	Intervention	1	232.82	< 0.001
	Age × species	1	0.41	0.525
	Age × intervention	1	136.59	< 0.001
	Species × intervention	1	1.36	0.249
	Age × species × intervention	1	0.90	0.348
	Residual	43		
Pieou_e Index	Age	1	114.38	< 0.001
	Species	1	1.13	0.294
	Intervention	1	168.79	< 0.001
	Age × species	1	0.68	0.416
	Age × intervention	1	99.16	< 0.001
	Species × intervention	1	0.30	0.584
	Age × species × intervention	1	1.23	0.274
	Residual	43		

**FIGURE 2:**
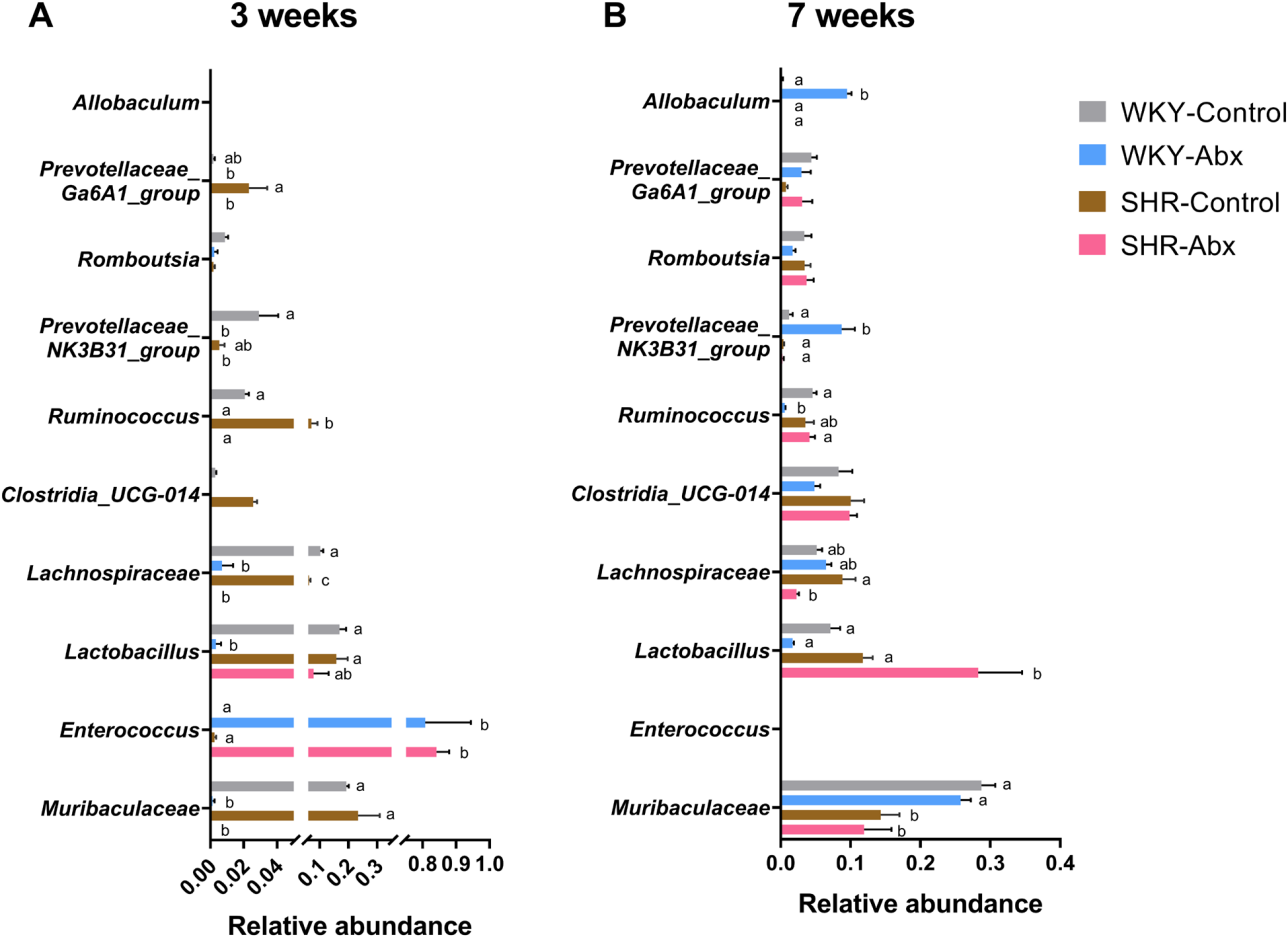
Comparisons of the relative abundance of gut microbiota at the genus level. (A) 3 weeks and (B) 7 weeks. *n* = 5 in each group at 3 weeks, *n* = 7–9 in each group at 7 weeks. Two‐way ANOVA was performed, and then simple effects of different groups were analyzed by one‐way ANOVA if the *p* value of interaction between age and group was less than 0.05. The small letters represent *p* < 0.05.

The top 10 abundances of the gut microbiota components at both phylum and genus levels were shown in Figure 1C. At the phylum level, *Firmicutes* predominated in the gut microbiota following ceftriaxone intervention at 3 weeks. By 7 weeks, the *Firmicutes*/*Bacteroidota* (F/B) ratio was significantly higher in the SHR group and markedly elevated in the SHR‐Abx group (Figure 1C). At the genus level, the gut microbiota composition is depicted in Figure 1C, with detailed comparisons of microbial relative abundance in Figure 2 and Table 2. At 3 weeks, ceftriaxone intervention led to a substantial increase in *Enterococcus* relative abundance, reaching approximately 80%, while its presence was suppressed at 7 weeks in both WKY and SHR. At 7 weeks, compared to the WKY‐Control group, the WKY‐Abx group exhibited a significant decrease in *Ruminococcus* and increases in *Prevotellaceae_NK3B31_group* and *Allobaculum*. In SHR, early life ceftriaxone treatment resulted in an increased relative abundance of *Lactobacillus* and a decreased abundance of *Lachnospiraceae*. Moreover, the relative abundance of *Muribaculaceae* was significantly lower in SHR compared to WKY, despite the antibiotic intervention. As for another gut microbiota, *Clostridia_UCG‐014*, no interaction was found between age and group, but the main effect of different groups was significant (Table 2), where its relative abundance was higher in SHR compared to WKY (Figure 2).

**TABLE 2:** ANOVA table of gut microbiota abundance at genus level.

Index	Source of variation	Df	*F*	*p*
*Allobaculum*	Age	1	98.84	< 0.001
	Group	3	96.81	< 0.001
	Age × group	3	97.06	< 0.001
	Residual	43		
*Prevotellaceae_Ga6A1_group*	Age	1	9.40	0.004
	Group	3	0.29	0.833
	Age × group	3	3.24	0.031
	Residual	43		
*Romboutsia*	Age	1	27.70	< 0.001
	Group	3	0.96	0.419
	Age × group	3	0.90	0.449
	Residual	43		
*Prevotellaceae_NK3B31_group*	Age	1	5.46	0.024
	Group	3	6.65	0.001
	Age × group	3	9.90	< 0.001
	Residual	43		
*Ruminococcus*	Age	1	1.97	0.168
	Group	3	11.29	< 0.001
	Age × group	3	6.69	0.001
	Residual	43		
*Clostridia_UCG‐014*	Age	1	63.84	< 0.001
	Group	3	3.03	0.040
	Age × group	3	1.22	0.313
	Residual	43		
*Lachnospiraceae*	Age	1	3.18	0.081
	Group	3	17.07	< 0.001
	Age × group	3	8.87	< 0.001
	Residual	43		
*Lactobacillus*	Age	1	0.73	0.398
	Group	3	10.03	< 0.001
	Age × group	3	7.68	< 0.001
	Residual	43		
*Enterococcus*	Age	1	220.45	< 0.001
	Group	3	73.12	< 0.001
	Age × group	3	73.07	< 0.001
	Residual	43		
*Muribaculaceae*	Age	1	18.58	< 0.001
	Group	3	12.01	< 0.001
	Age × group	3	10.77	< 0.001
	Residual	43	98.84	< 0.001

Overall, these results suggest that early life ceftriaxone intervention has lasting effects on gut microbial diversity and composition. Alterations in gut microbiota diversity, the F/B ratio, and specific gut microbes may play a role in the development of ADHD in males.

### 3.2 Gut Microbiota Perturbation in Early Life Resulted in Neuroinflammation, Peripheral Immune Dysfunction in Juvenile

**FIGURE 3:**
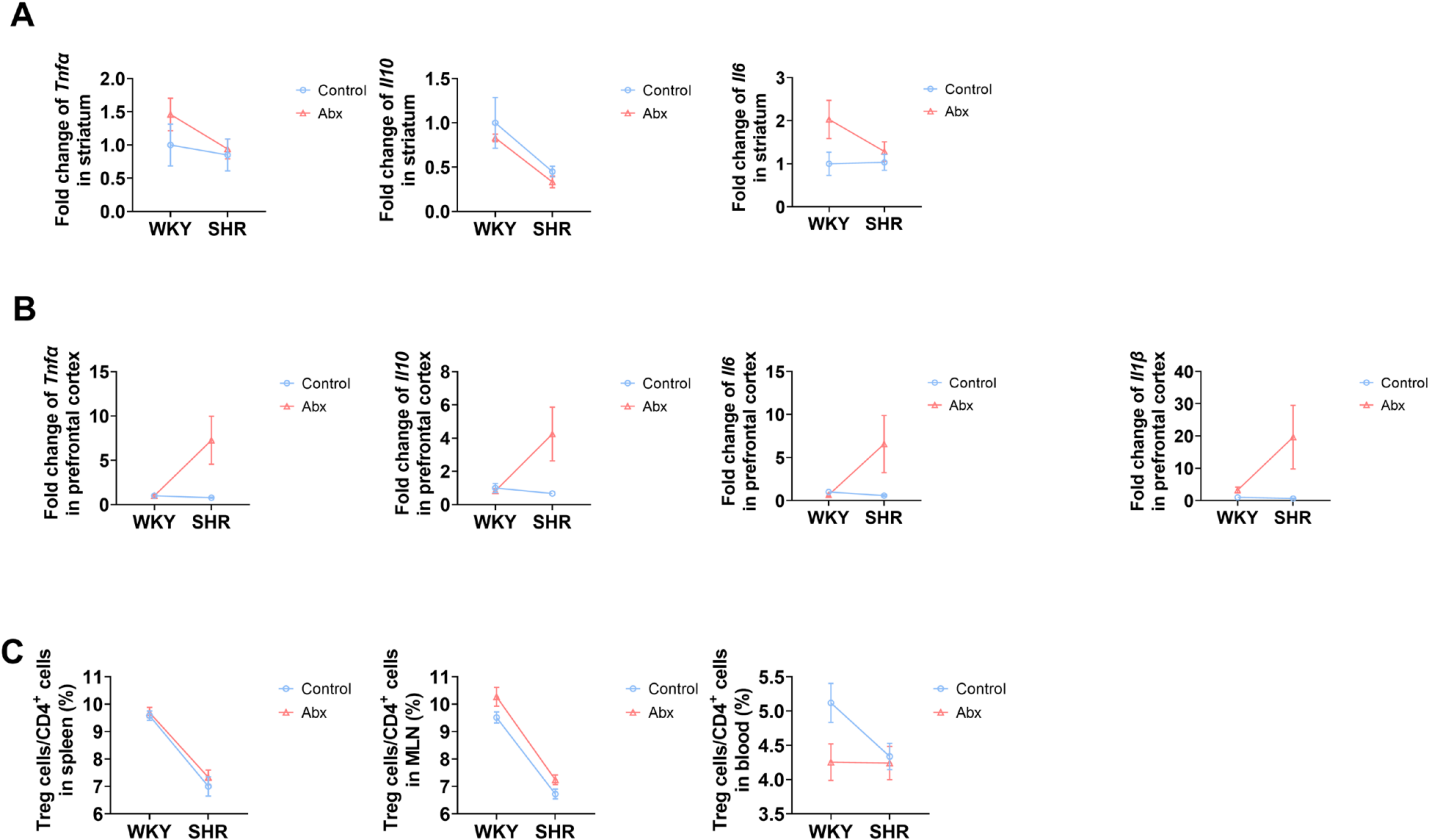
Effects of gut microbiota perturbation in early life on inflammation at 7 weeks. (A, B) The mRNA expression of cytokines in the brain, *n* = 5–7 in each group. (C) Percentage of Treg cells/CD4^+^ T cells in MLN, spleen, and blood, *n* = 7–10 in each group. Abx, ceftriaxone group. Two‐way ANOVA was conducted, and if the *p* value of the interaction is less than 0.05, simple effects of the intervention were analysed with *t* test corrected by Bonferroni.

In the striatum, interleukin‐10 (IL‐10) mRNA expression was significantly lower in SHR compared to WKY (Figure 3A, Table 3, *p* = 0.001). Ceftriaxone intervention resulted in a marginal increase in IL‐6 mRNA expression in both WKY and SHR (Figure 3A, Table 3, *p* = 0.068). However, in the prefrontal cortex, there was an interaction between species and intervention in tumour necrosis factor‐alpha (TNF‐α) and IL‐10 mRNA expression (Figure 3B, Table 3, *p* = 0.017 and 0.022, respectively). Overall, early life ceftriaxone treatment increased tumour necrosis factor‐alpha (TNF‐α) and IL‐10 mRNA expression in SHR (Figure 3B *p* = 0.004 and 0.009, respectively), and increased IL‐1β mRNA expression in both WKY and SHR (Figure 3B, Table 3, *p* = 0.031).

**TABLE 3:** ANOVA table of inflammation indices.

Index	Source of variation	Df	*F*	*p*
*Tnfa* in striatum	Species × intervention	1	0.55	0.466
	Species	1	1.81	0.195
	Intervention	1	1.22	0.282
	Residual	19		
*Il10* in striatum	Species × intervention	1	0.04	0.847
	Species	1	15.21	0.001
	Intervention	1	1.14	0.299
	Residual	19		
*Il6* in striatum	Species × intervention	1	1.38	0.256
	Species	1	1.15	0.296
	Intervention	1	3.74	0.068
	Residual	19		
*Tnfa* in prefrontal cortex	Species × intervention	1	6.86	0.017
	Species	1	5.97	0.025
	Intervention	1	6.87	0.017
	Residual	18		
*Il10* in prefrontal cortex	Species × intervention	1	6.31	0.022
	Species	1	4.28	0.053
	Intervention	1	5.17	0.035
	Residual	18		
*Il6* in prefrontal cortex	Species × intervention	1	4.34	0.052
	Species	1	3.27	0.087
	Intervention	1	3.45	0.080
	Residual	18		
*Il1β* in prefrontal cortex	Species × intervention	1	3.43	0.081
	Species	1	3.16	0.093
	Intervention	1	5.52	0.031
	Residual	18		
Treg cells in spleen	Species × intervention	1	0.17	0.684
	Species	1	90.90	< 0.001
	Intervention	1	0.73	0.402
	Residual	27		
Treg cells in MLN	Species × intervention	1	0.22	0.643
	Species	1	132.30	0.001
	Intervention	1	6.36	0.018
	Residual	27		
Treg cells in blood	Species × intervention	1	2.26	0.144
	Species	1	2.39	0.133
	Intervention	1	3.51	0.072
	Residual	28		

**FIGURE 4:**
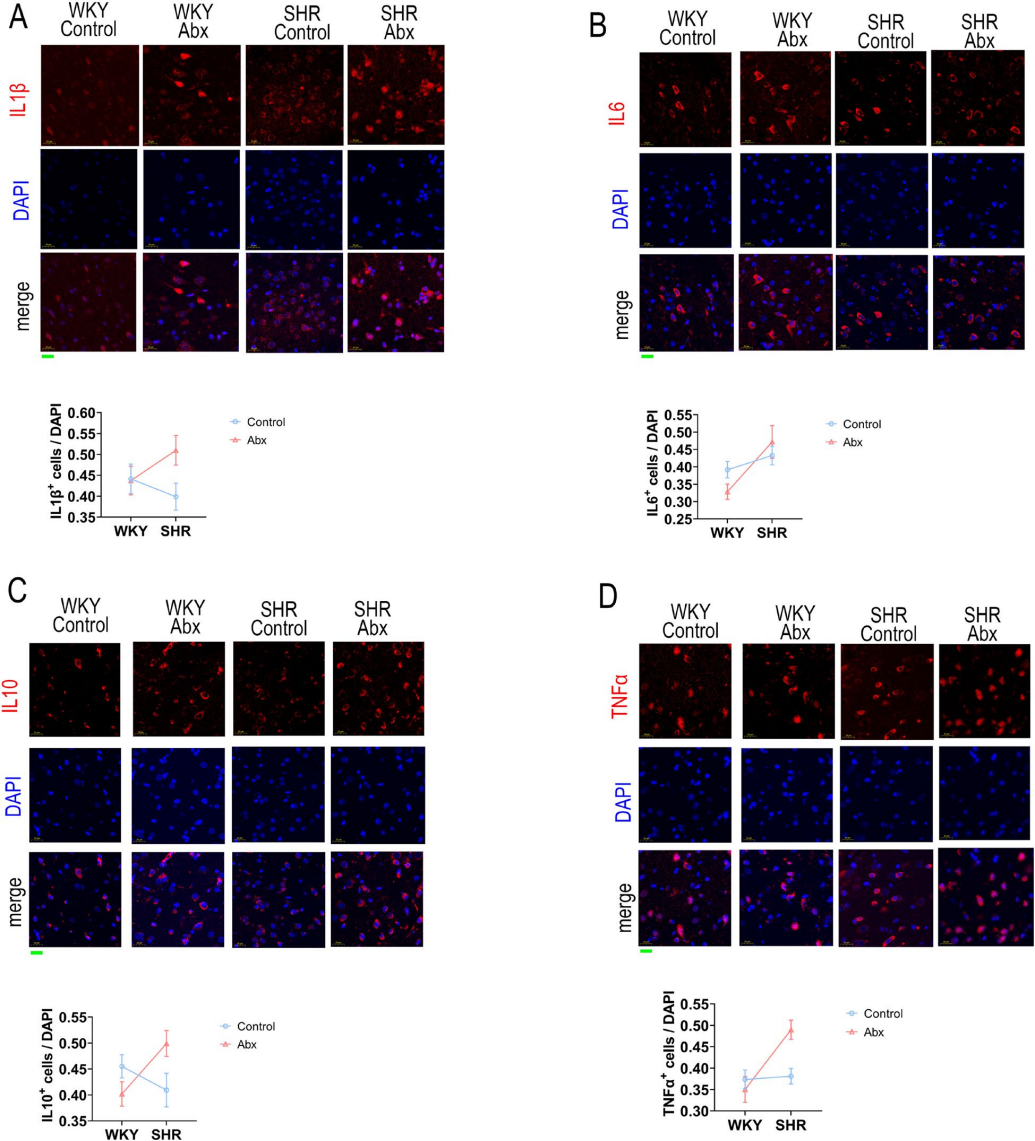
IF staining for (A) IL‐1β, (B) IL‐6, (C) IL‐10 and (D) TNF‐α. The ratio of positive cells/DAPI in each 40× view was calculated. A total of 8 views in each group were included (*n* = 8). The green bar in the lower left corner represents 20 μm. Abx, ceftriaxone group. Two‐way ANOVA was conducted, and if the *p* value of the interaction is less than 0.05, simple effects of the intervention were analysed with *t* test corrected by Bonferroni.

The expression of these cytokines was further investigated by IF staining (Figure 4, Table 4). Accordingly, there was also an interaction between species and intervention in TNF‐α and IL‐10 expression (Figure 4, Table 4, *p* = 0.010 and 0.009, respectively). TNF‐α and IL‐10 expression increased in SHR (Figure 4, *p* = 0.006 and 0.043, respectively). However, SHR had a higher expression of IL‐6 than WKY, despite the antibiotic intervention (Figure 4, Table 4, *p* = 0.007).

**TABLE 4:** ANOVA table of cytokines expression by IF staining in the prefrontal cortex.

Cytokines	Factor	Df	*F*	*p*
IL‐1β	Species	1	0.19	0.665
	Intervention	1	2.43	0.130
	Species × intervention	1	2.82	0.104
	Residual	28		
IL‐6	Species	1	8.52	0.007
	Intervention	1	0.15	0.703
	Species × intervention	1	2.62	0.117
	Residual	28		
IL‐10	Species	1	0.98	0.330
	Intervention	1	0.50	0.487
	Species × intervention	1	7.54	0.010
	Residual	28		
TNF‐α	Species	1	9.75	0.004
	Intervention	1	3.27	0.081
	Species × intervention	1	7.88	0.009
	Residual	28		

The proportion of Treg cells in CD4^+^ T cells was lower in both the spleen and MLN of SHR compared to WKY (Figure 3C, Table 3, *p* < 0.001 and *p* = 0.001, respectively). Ceftriaxone intervention significantly reduced the proportion of Treg cells in MLN of WKY and SHR (Figure 3C, Table 3, *p* = 0.018), and slightly reduced its proportion in blood of WKY and SHR (Figure 3C, Table 3, *p* = 0.072).

Overall, these results suggest that gut microbiota perturbation in early life can induce neuroinflammation in the brain and disrupt peripheral immune responses in both WKY and SHR.

### 3.3 Gut Microbiota Perturbation in Early Life Induced Juvenile ADHD‐Like Behaviours

**FIGURE 5:**
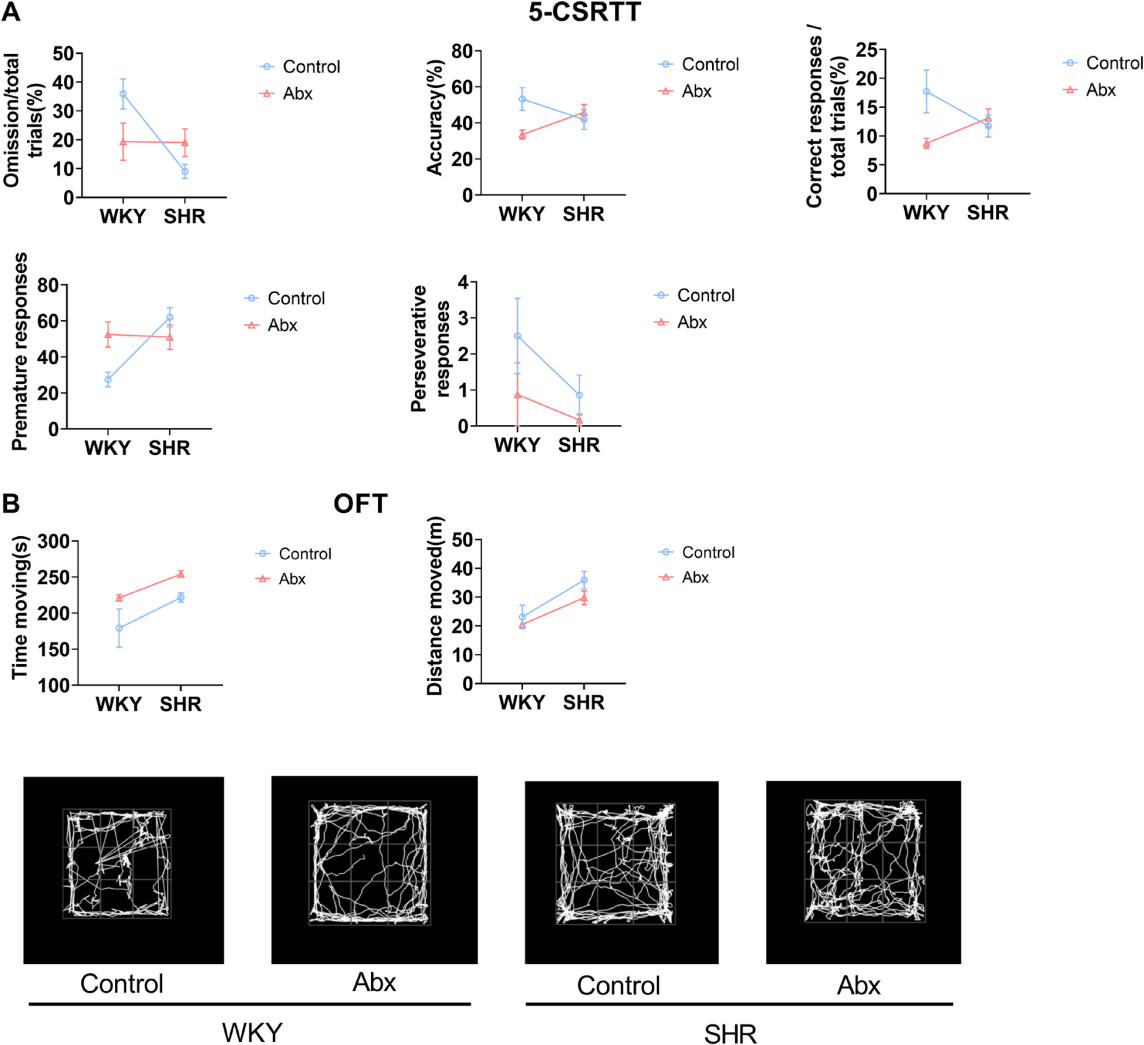
Effects of gut microbiota perturbation in early life on juvenile ADHD‐like behaviours. (A) 5‐CSRTT, *n* = 4–8 in each group. (B) OFT (upper) and representative trace map (lower), *n* = 6–9 in each group. Abx, antibiotic group. Two‐way ANOVA was conducted, and if the *p* value of the interaction is less than 0.05, simple effects of the intervention were analysed with t test corrected by Bonferroni.

In the 5‐CSRTT, there were interactions between species and interactions in terms of omission, accuracy, correct response, and premature response (Table 5). However, early life ceftriaxone intervention decreased accuracy and correct responses and increased premature responses in WKY (*p* = 0.020, *p* = 0.008, and *p* = 0.040, respectively), indicating the development of inattention and impulsivity (Figure 5A). In the OFT, SHR showed inherent hyperactivity compared to WKY, as reflected by significantly higher time moving and distance moved in the OFT (Figure 5B, Table 5, *p* = 0.003 and *p* < 0.001, respectively). Notably, early life ceftriaxone intervention increased the time moving in the OFT in both WKY and SHR (Figure 5B, Table 5, *p* = 0.004). These results suggest that early life gut microbiota perturbation may induce ADHD‐like behaviours in WKY and severe hyperactivity in SHR.

**TABLE 5:** ANOVA table of behavioural tests.

Index	Source of variation	Df	*F*	*p*
Omission in 5‐CSRTT	Species × intervention	1	6.02	0.023
	Species	1	6.33	0.020
	Intervention	1	0.37	0.550
	Residual	21		
Accuracy in 5‐CSRTT	Species × intervention	1	6.37	0.020
	Species	1	0.01	0.921
	Intervention	1	2.82	0.108
	Residual	21		
Correct response in 5‐CSRTT	Species × intervention	1	7.64	0.012
	Species	1	0.18	0.674
	Intervention	1	4.15	0.055
	Residual	21		
Premature responses in 5‐CSRTT	Species × intervention	1	7.20	0.014
	Species	1	6.05	0.023
	Intervention	1	1.09	0.309
	Residual	21		
Perservative responses in 5‐CSRTT	Species × intervention	1	0.39	0.542
	Species	1	2.44	0.133
	Intervention	1	2.36	0.139
	Residual	21		
Time moving in OFT	Species × intervention	1	0.17	0.684
	Species	1	10.56	0.003
	Intervention	1	10.27	0.004
	Residual	26		
Distance moved in OFT	Species × intervention	1	0.46	0.503
	Species	1	17.63	< 0.001
	Intervention	1	2.74	0.110
	Residual	26		

### 3.4 Network Correlation of the Gut Microbiota, Biochemical Indies, and Behavioural Performance

**FIGURE 6:**
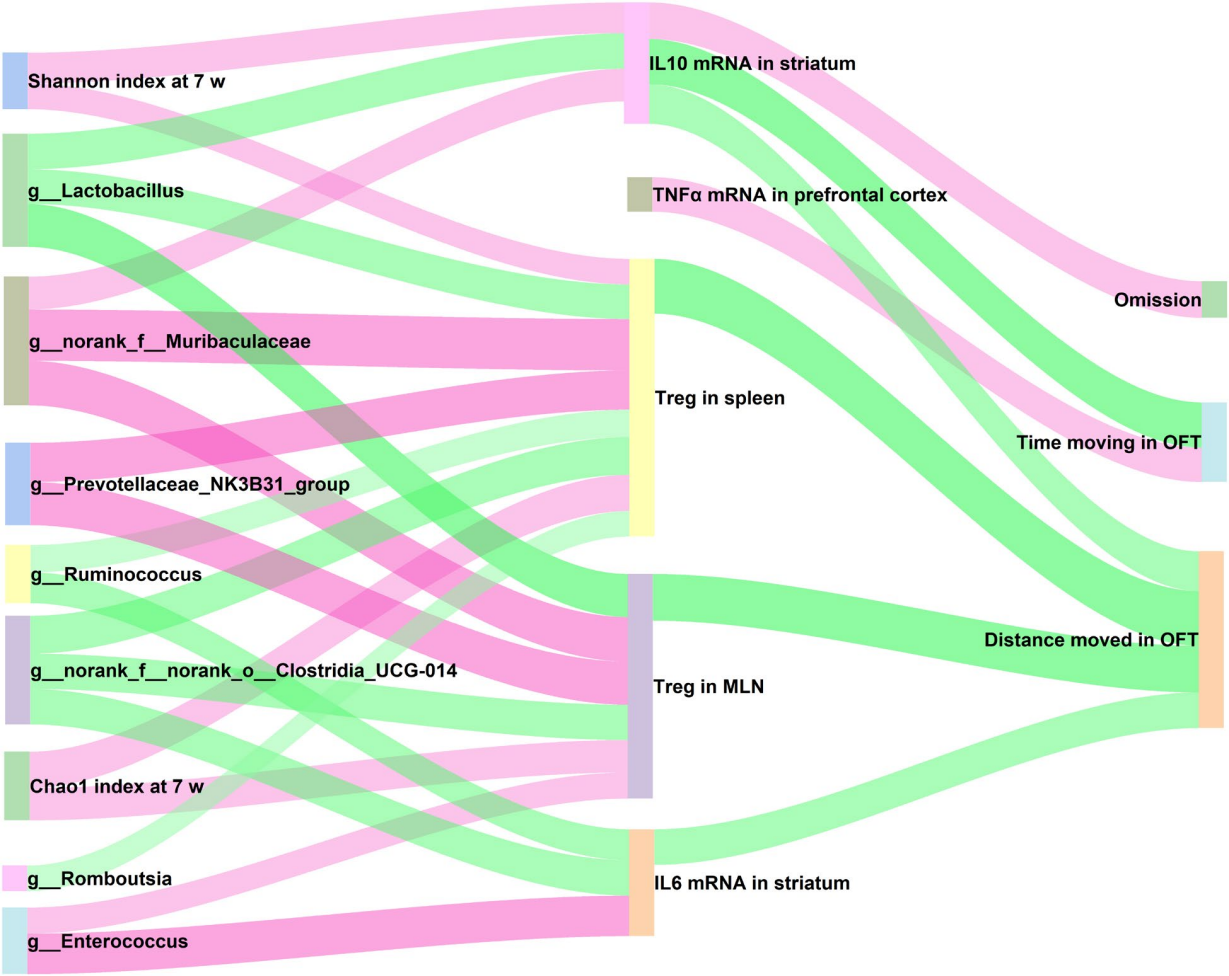
Pearson correlation of gut microbiota, biochemical indices and behavioural tests. Only the data of *p* < 0.05 was shown. Red line means positive correlation, and green line means negative correlation. The width of the line represents the absolute value of the *r*. The IF staining results were not one‐to‐one matched with other results, and thus were not included in the correlation analysis.

Network correlation analysis (Figure 6) reveals broad associations between Chao1 Index, Shannon Index, and key gut microbiota genera (*Lactobacillus*, *Prevotellaceae_NK3B31_group*, *Muribaculaceae*, and *Clostridia_UCG‐014*) with various biochemical indices and behavioral outcomes.

Specifically, *Lactobacillus* and *Clostridia_UCG‐014* were negatively associated, while *Prevotellaceae_NK3B31_group* and *Muribaculaceae* were positively associated with Treg cells in the spleen and MLN. These biomedical indices were, in turn, negatively associated with the distance moved in the OFT, a measure of hyperactivity. Besides, *Muribaculaceae* were positively associated with IL‐10 mRNA expression in the striatum, and *Clostridia_UCG‐014* were negatively associated with IL‐6 mRNA expression in the striatum. These two biomedical indices were then negatively associated with the distance moved in the OFT. Furthermore, *Muribaculaceae* alone were positively associated with IL‐10 mRNA expression in the striatum, which was then negatively associated with time moving in the OFT.

Consequently, it is inferred that those key gut microbes, especially *Muribaculaceae* and *Clostridia_UCG‐014*, may have the potential to contribute to ADHD‐like behaviours by influencing central and peripheral immune functions.

## 4 Discussion

In this study, we used ceftriaxone sodium to induce gut microbiota perturbation from birth to weaning in both male WKY and SHR, with microbial alterations persisting into the juvenile stage despite the absence of further intervention for the following 4 weeks. In the juvenile period, both central and peripheral inflammation were activated, which were associated with the emergence of ADHD‐like behaviours in WKY and SHR.

Many researches have investigated the differences in gut microbiota diversity and composition between children with ADHD and healthy controls, reporting reduced α and β diversity (Wang et al. 2024; Prehn‐Kristensen et al. 2018) and altered abundance of several specific microbial taxa (Wang et al. 2022). However, studies examining the association between gut microbiota perturbations in early life and ADHD in later life in animal models remain limited. A previous study by Qiu et al. (2022) revealed that perinatal exposure to low‐level polybrominated diphenyl ethers in rats contributed to hyperactivity and anxiety‐like behaviour in offspring at adulthood by disturbing the gut microbiota and altering serum metabolites, suggesting that gut microbiota perturbation may induce ADHD‐like behaviour. Furthermore, the present study is the first to demonstrate a relationship between gut microbiota perturbation in early life and ADHD‐like behaviours by using an established animal model. Specifically, WKY exhibited all three ADHD‐like behaviours after early life ceftriaxone intervention, and SHR displayed more severe hyperactivity and neuroinflammation. Thus, these findings reinforce the hypothesis that gut microbiota in early life is a contributing factor in the development of ADHD.

This study further observed that early life antibiotic treatment had lasting effects, including reduced gut microbiota diversity, an increased F/B ratio, and altered microbial composition, consistent with our previous findings (Cheng et al. 2019). Specifically, the genera *Lactobacillus*, *Prevotellaceae_NK3B31_group*, and *Clostridia_UCG‐014* at juvenile were identified to be highly positively associated with ADHD, while *Muribaculaceae* was highly negatively associated. We found that the relative abundance of *Muribaculaceae* was lower, while that of *Clostridia_UCG‐014* was higher in juvenile SHR compared to WKY, despite the antibiotic intervention. Previous studies have shown that *Muribaculaceae* can produce short‐chain fatty acids (SCFA) and succinate (Ormerod et al. 2016; Smith et al. 2021), and may mitigate neuroinflammation by modulating host metabolism (Zhao et al. 2022). Conversely, *Clostridia_UCG‐014* is considered a conditional pathogen and pro‐inflammatory taxon (Liu et al. 2024), and has been reported to proliferate in the gut of mice with cognitive decline induced by circadian rhythm disorder (Song et al. 2024), as well as in patients with Parkinson's disease (Pavan et al. 2023). In line with these results, our research suggested that *Clostridia_UCG‐014* might contribute to the onset of ADHD, while *Muribaculaceae* appeared to exert a protective effect. Regarding *Lactobacillus* and *Prevotellaceae_NK3B31_group*, their relative abundances significantly increased at the juvenile stage in both SHR and WKY after early life antibiotic intervention. Metabolites produced by these two genera, such as indole‐3‐acetate and SCFA, have been reported to modulate the MGB axis (Wei et al. 2024; Li et al. 2022). However, their specific roles in ADHD pathophysiology still need further investigation due to the limited available literature.

Neuroinflammation has been proposed as a risk factor of ADHD (Dunn et al. 2019), as it can induce glial activation (Réus et al. 2015), increase oxidative stress (Hassan et al. 2016), reduce neurotropic support (Sen et al. 2008), and alter neurotransmitter function (Kronfol and Remick 2000), thereby influencing brain development. An increased number of activated microglial cells and elevated TNF‐α expression levels have been found in the brain of SHR (Fang et al. 2023). Consistently, our study also observed elevated mRNA expression levels of several cytokines, including IL‐1β, IL‐6, IL‐10, and TNF‐α in the prefrontal cortex of SHR after gut microbiota perturbation in early life. These findings are consistent with an earlier study reporting elevated cerebrospinal fluid levels of the pro‐inflammatory cytokine TNF‐β and reduced levels of the anti‐inflammatory cytokine IL‐4 in individuals with ADHD (Mittleman et al. 1997). These results indicated that ceftriaxone‐induced gut microbiota perturbation in early life could lead to a sustained elevation of inflammatory cytokines through modulating the MGB axis, thereby exacerbating neuroinflammation in the ADHD animal model.

Treg cells contribute to immune homeostasis by inhibiting the function of antigen‐presenting cells and effector cells, or releasing anti‐inflammatory cytokines such as IL‐10 and TGF‐β (Josefowicz et al. 2012; Barbi et al. 2014). Treg cells have also been shown to exert neuroprotective effects in the context of depression (Gao et al. 2023). In our study, the percentage of Treg cells in the spleen and MLN of SHR was significantly lower than that in WKY, and early life ceftriaxone intervention decreased the percentage of Treg cells in MLN in both WKY and SHR. These results indicated that Treg cells may be involved in the pathophysiology of ADHD and could play a protective role. However, a case–control study reported significantly higher levels of peripheral Treg cells in children with ADHD compared to healthy children (Cetin et al. 2022). Kozłowska et al. reported that elevated levels of cytokines, chemokines, oxidative stress markers, and medial prefrontal cortex alterations were found only in juvenile SHRs (5 weeks old), but not in maturating SHRs (10 weeks old), suggesting that increased steroid hormones in older SHRs may present a compensatory mechanism (Kozłowska et al. 2019). These findings imply that age may be a critical factor in studies of ADHD. Therefore, the age‐dependent role of Treg cells in ADHD warrants further investigation.

Finally, the primary strength of this study lies in its novel demonstration of the relationship between ADHD and gut microbiota, particularly during early developmental stages, using an animal model. By restricting the study to juvenile animals, the model more accurately reflects childhood ADHD and minimizes potential confounding effects of hypertension in SHR. However, this study has two main limitations. Firstly, only male WKY and SHR were included. Although ADHD is more prevalent in males, previous studies have reported sex‐related differences in both ADHD symptoms and gut microbiota composition (Ramtekkar et al. 2010; Davis et al. 2017). Secondly, this study solely focused on the immune pathway within the MGB axis, without addressing other potential mediators, such as microbial metabolites, monoamine neurotransmitters (e.g., dopamine) involved in neural and endocrine signaling. Moreover, previous studies have demonstrated interactions between dopamine receptors and neuroinflammation, showing that various dopamine receptor subtypes are expressed in microglia and astrocytes (Xia et al. 2019; Pocock and Kettenmann 2007). Future studies should comprehensively investigate sex‐specific differences in gut microbiota and the involvement of additional mediators within the MGB axis in ADHD, employing multi‐omics and neuroimaging approaches.

## 5 Conclusion

After early life gut microbiota perturbation by ceftriaxone treatment, WKY exhibited behavioural characteristics resembling those of SHR, while SHR showed exacerbated hyperactivity and neuroinflammation. This early‐life gut microbiota disturbance persisted into the juvenile stage and influenced immune‐related pathways within the MGB axis. These alterations included activation of peripheral immune responses and neuroinflammation, both of which contribute to the onset and exacerbation of ADHD‐like behaviours in juvenile WKY and SHR. This study highlights potential mechanisms through which early life gut microbiota disturbances may contribute to the development of juvenile ADHD, thereby providing a foundation for novel therapeutic strategies. These strategies may include interventions targeting gut microbiota, such as probiotics and prebiotics, to prevent or treat ADHD, as well as approaches aimed at modulating immune responses to manage the condition.

## Author Contributions

**Yang Yang:** investigation, methodology, writing – original draft; **Simou Wu:** methodology; **Jianxiu Liu:** investigation; **Kai Wang:** investigation; **Yating Luo:** investigation; **Jinxing Li:** methodology; **Zhimo Zhou:** investigation; **Fang He:** supervision; **Ruyue Cheng:** methodology, supervision, writing – review and editing.

## Conflicts of Interest

The authors declare no conflicts of interest.

## Supporting information


**Data S1:** mbt270255‐sup‐0001‐Supinfo1.docx.

## Data Availability

The data that support the findings of this study are available from the corresponding author upon reasonable request.
