# Supplementary material for: Early‐Life Ceftriaxone‐Induced Gut Microbiota Perturbation Persistently Exacerbates Juvenile ADHD‐Like Behaviours via Immune Dysfunction in SHR/WKY Rats
Source: Microb Biotechnol. 2025 Oct 23;18(10):e70255. doi: 10.1111/1751-7915.70255 (PMC12547482; doi:10.1111/1751-7915.70255)
Supplement: Supplementary file 1 — Data S1: mbt270255‐sup‐0001‐Supinfo1.docx. [file MBT2-18-e70255-s001.docx]

**Supplementary materials**

Table S 1 Parameters of 5-CSRTT test

| Test day | Stimulus duration (s) | Limited  hold (s) | Intertrial interval (s) | Timeout (s) |
| --- | --- | --- | --- | --- |
| 1-5 | 10 | 10 | 5 | 5 |
| 6 | 8 | 9 | 5 | 5 |
| 7 | 6 | 8 | 5 | 5 |
| 8 | 5 | 7 | 5 | 5 |
| 9 | 4 | 6 | 5 | 5 |
| 10 | 3 | 5 | 5 | 5 |
| 11 | 2 | 5 | 5 | 5 |
| 12-16 | 1 | 5 | 5 | 5 |
| 17 | 1 | 5 | 5 | 5 |

Table S 2 The reverse-transcription PCR protocol

| Step | Parameter |
| --- | --- |
| Priming | 5 min at 25℃ |
| Reverse transcription | 20 min at 46℃ |
| RT inactivation | 1 min at 95℃ |
| Final step | Hold at 4℃ |

Table S 3 The qPCR protocol

| Step | Parameter |
| --- | --- |
| Polymerase activation and DNA denaturation | 30 s at 98℃ |
| Denaturation | 15 s at 98℃ |
| Extension | 10 s at 60℃ (*Tjp1, Ocln, Drd1, Th, Gabra1, Adra2a, Arbp, Il10*) |
|  | 20 s at 60℃ (*Cldn5, Drd2, Gabbr1, Adra2c, Gapdh, Actb*) |
|  | 30 s at 60℃ (*Cldn1, Htr1a, Htr2a, Il1β, Il6, Tnfα*) |
| Plate read | - |
| Cycles | 40 |
| Melt curve analysis | 65-95℃ (0.5℃ increments) |

Table S 4 The primer sequences

| Gene | Forward (5’-3’) | Reverse (5’-3’) |
| --- | --- | --- |
| *Tjp1* | TGTGGGTTTGCGACTAGCTG | TCTTTGGCTGCAGGGCTATC |
| *Ocln* | CGACGAGGTCAACAAAGAGC | CAGCAGCAGCCATGTACTCT |
| *Cldn1* | TGGGGACAACATCGTGACTG | ATCAAGGCTCTGGTTGCCTG |
| *Cldn5* | GGGCGTCCAGAGTTCAGTTT | TCCCGCCCTTAGACGTAGTT |
| *Il1β* | GGCTTCCTTGTGCAAGTGTC | AAGGGCTTGGAAGCAATCCT |
| *Il6* | TCAACTCCATCTGCC | GGGTGGTATCCTCTGT |
| *Il10* | TTGAACCACCCGGCATCTAC | CCAAGGAGTTGCTCCCGTTA |
| *Tnfα* | CTGGCGTGTTCATCCGTTCT | GTTGGACCCAGAGCCACAAT |
| *Actb* | GCAGATGTGGATCAGCAAGC | AGAAAGGGTGTAAAACGCAGC |
| *Arbp* | CACTCGCTTCCTAGAGGGTGT | TGAGGCAACAGTCGGGTAGC |
| *Gapdh* | GCCGCATCTTCTTGTGCAG | ATGAAGGGGTCGTTGATGGC |
